# Efficacy of Sublingual Immunotherapy with *Dermatophagoides farinae* Extract in Monosensitized and Polysensitized Patients with Allergic Rhinitis: Clinical Observation and Analysis

**DOI:** 10.1155/2015/187620

**Published:** 2015-04-27

**Authors:** Chen-Xia Xu, Miao-Lian Zhang, Bi-Zhou Li, Ying He, Ze-Hong Zou, Qiu-Rong Wu, Ai-Lin Tao, He Lai, Jin-Lu Sun

**Affiliations:** ^1^Guangdong Provincial Key Laboratory of Allergy & Clinical Immunology, The State Key Laboratory of Respiratory Disease, The Second Affiliated Hospital of Guangzhou Medical University, 250 Changgang Road East, Guangzhou 510260, China; ^2^Department of Allergy, Peking Union Medical College Hospital, Chinese Academy of Medical Sciences, No. 1, Shuai Fu Yuan, East District, Beijing 100730, China

## Abstract

*Aim*. To investigate differences in the efficacy of sublingual immunotherapy with *Dermatophagoides farinae* drops in monosensitized and polysensitized allergic rhinitis patients. *Methods*. The patients enrolled in the study were treated for more than one year by sublingual immunotherapy (SLIT) using *Dermatophagoides farinae* drops and were divided into a monosensitized group (*n* = 20) and a polysensitized group (*n* = 30). Total nasal symptom scores of patients before and after SLIT were analyzed to evaluate the curative effect. The phylogenetic tree of dust mite allergens as well as other allergens that were tested by skin prick test was constructed to help the analysis. *Results*. There was no significant difference in the efficacy of SLIT between dust mite monosensitized and polysensitized patients. *Conclusions*. Both dust mite monosensitized and polysensitized patients could be cured by SLIT using *Dermatophagoides farinae* drops. This study provides a reference for the selection of allergens to be used in immunotherapy for polysensitized AR patients.

## 1. Introduction

Allergic rhinitis (AR) is a global health problem that seriously affects patients' daily life [1]. Epidemiological data indicates that AR and asthma are the same airway disease. There is a great desire for treatments of AR that can also prevent and control the occurrence and progress of bronchial asthma [2]. Some studies have shown that patients with reactivity to multiple allergens accounted for a large proportion of the allergic population [3–5] and reactivity to house dust mite (HDM) is the most prevalent allergen seen in the patients with asthma and AR [6]. Treatments for AR include avoidance, symptomatic treatment, and allergen immunotherapy. Allergen-specific immunotherapy (ASIT) is currently the only available treatment able to moderate the typical symptoms of AR [7]. However, conventional subcutaneous ASIT requires 30 to 80 injections in three to five years, which leads to poor compliance by the patients. In contrast, sublingual immunotherapy (SLIT) offers a noninvasive, nonpainful, and more convenient treatment. We analyzed the differences in the curative effect of treatment with dust mite SLIT between monosensitized and polysensitized patients in order to help us build a foundation for further development of representative allergen-specific immunotherapy.

## 2. Materials and Methods

### 2.1. Study Population

All patients who consulted the Allergy Department in the Second Affiliated Hospital of Guangzhou Medical University between January 2008 and August 2012 were consecutively enrolled. The inclusion criteria were as follows: (1) aged between 4 and 60; (2) diagnosed with moderate to severe dust mite AR through medical interviews and clinical symptoms by allergists according to criteria described by Allergic Rhinitis and its Impact on Asthma (ARIA) [8]; (3) having positive skin prick test (SPT) to dust mite allergens; (4) having dust mite specific IgE higher than 0.35 kU/L; (5) having duration of SLIT at least one year; (6) willing to accept follow-up evaluation and stop the treatment for 1 to 2 years; (7) not having acute or chronic sinusitis, organic nasal disease, nonallergic autoimmune disease, malignant tumor, chronic infection, or mental disorder. Written informed consent was obtained from all subjects. According to the results of the SPT and allergen-specific IgE antibodies, patients were divided into two groups that were either monosensitized to dust mite only or polysensitized to dust mite as well as other allergens.

### 2.2. Skin Prick Test

SPT was performed by trained nurses on the volar aspect of the subjects' forearms with 50 mg/mL to 200 mg/mL of inhalant allergen extracts using standard procedures. None of the patients had taken medications that might interfere with SPT two weeks before the test. SPT was performed using the following inhalant allergen extracts prepared in a sterile environment followed by toxicity and potency evaluation according to an in-house standard protocol as described [9]: 67 mg/mL animal dander (duck, chicken, rabbit, porcine, and goose), 50 mg/mL spring pollen (*Acacia confusa* Merr., pine tree, cedar,* Broussonetia papyrifera*,* Myrica rubra*, Chinese Mulberry, and* Livistona chinensis*), 50 mg/mL summer pollen (maize,* Casuarina equisetifolia*,* Melia azedarach*, and* Eucalyptus camaldulensis*), 50 mg/mL autumn pollen (*Mallotus apelta*,* Humulus scandens*, mugwort,* Vitex negundo,* and* Sesbania cannabina* Pers.), 50 mg/mL winter pollen (*Melaleuca leucadendra* and* Bauhinia blakeana* Dunn.), 50 mg/mL spiny amaranth (*Amaranthus spinosus* L.) pollen, 50 mg/mL cockroaches, 50 mg/mL moths, 50 mg/mL bees, 67 mg/mL silk, 50 mg/mL mites (*Dermatophagoides pteronyssinus* and* Dermatophagoides farinae*), 200 mg/mL house dust, 67 mg/mL padding, 67 mg/mL cat hair, and 67 mg/mL dog hair. Buffer solution was used as a negative control and 10 mg/mL histamine dihydrochloride (ALK-Abello, Hørsholm, Denmark) was used as positive control concurrently with SPT. Each drop of allergen extract solution was approximately 15 *μ*L and was pricked onto the skin with a sterile lancet (ALK-Abello, Hørsholm, Denmark). The distance between the location of the positive control and the locations of the allergen extracts was more than 4 cm. SPT results were recorded after 15 min and the wheals were outlined and transferred to paper with transparent tape. The mean wheal diameter (MD) was calculated according to the formula (*D* + *d*)/2, where *D* was the largest longitudinal diameter and *d* was the largest transverse diameter. The mean value of the wheals was calculated and considered positive if at least 3 mm in diameter.

### 2.3. Determination of Allergen-Specific Antibodies

Serum allergen-specific IgE antibody was measured using the ImmunoCAP technology-UNICAP 100 (Pharmacia AB Diagnostics, Uppsala, Sweden). A positive result was defined as ≥0.35 kU/L.

### 2.4. Evaluation of Nasal Symptoms

Nasal symptoms were recorded before and after the therapy using questionnaires and a total nasal symptoms score was calculated [10] (Table 1). The therapy effectiveness was calculated for each patient as (the symptom score before therapy − symptom score after therapy) × 100%/symptom score before therapy. The patients were discriminated into three classes according to their therapy effectiveness: therapy effectiveness being more than 65% was regarded as markedly effective, 65%~26% effective, and less than 26% ineffective.

### 2.5. Sublingual Immunotherapy

**Figure 1:**
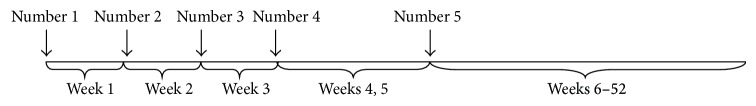
Dosage regimen of SLIT using* Dermatophagoides farinae* drops. Drops number 1 to number 3 with daily doses of 1, 2, 3, 4, 6, 8, or 10 drops were administered for the first three weeks, followed by daily maintenance doses using 3 drops of number 4 or number 5 in the following two weeks and after the 6th week, respectively.

The treatment course was at least 12 months and was performed with* Dermatophagoides farinae* drops (Figure 1). The concentration of* Dermatophagoides farinae* drops number 1 to number 5 was 1 *μ*g/mL, 10 *μ*g/mL, 100 *μ*g/mL, 333 *μ*g/mL, and 1000 *μ*g/mL, respectively. These drops include increasing therapeutic doses and a maintenance dose. Number 1 to number 4 were the increasing doses and number 5 was the maintenance dose for patients above 14 years of age. For patients under the age of 14, number 1 to number 3 were the increasing doses and number 4 was the maintenance dose. Daily doses of drops number 1 through number 3 were administered as 1, 2, 3, 4, 6, 8, or 10 drops every 7 days, followed by maintenance doses using 3 drops of number 4 and number 5. Drops were instructed to be kept under the tongue for 2 min before being swallowed.

### 2.6. Statistical Method

Qualitative data were analyzed using statistical software SPSS 13.0 (SoftPol, IBM, USA). The Kruskal-Wallis (KW) test was used for analysis of differences in curative effects. Statistical significance was assumed at *P* < 0.05.

### 2.7. Phylogenetic Tree of Der p 10

Amino acid sequences of Der p 10 and other homological allergens were searched in UniprotKB (http://www.uniprot.org/). Identity comparison was made with ClustalX 1.83 and the phylogenic tree was generated by MEGA 4.1.

## 3. Results and Discussion

### 3.1. Symptom Scores

50 patients were enrolled after screening and all of them lived in an urban environment. The curative effects of patients treated with sublingual immunotherapy for more than 1 year were analyzed. 22 of 50 patients reported markedly effective relief of symptoms, 15 cases reported effective relief, and 13 cases reported ineffective relief, with a total of 74% of patients reporting a relief of their symptoms (markedly effective + effective).

### 3.2. Distribution of Sensitized Patients to Specific Allergens

After combining their medical records with clinical manifestations, SPT, and serological testing results, patients were divided into two groups: monosensitized to only dust mite or polysensitized to multiple allergens. We found that 30 of the 50 patients (60%) were polysensitized to a variety of allergens other than dust mite. Accordingly, we further analyzed the rate of positive sensitivity of these patients to other allergens (Table 2). The most common positive allergens were cockroach (83.0%), winter pollen (70.0%), and silk (63.0%). In consideration of potential cross-reactivity between dust mite and other allergens [11], we further analyzed whether SLIT with* Dermatophagoides farinae* drops was able to elicit the same effectiveness in polysensitized patients as in monosensitized patients.

### 3.3. Comparison of SLIT Effectiveness between Monosensitized and Polysensitized Patients

Differences in the efficiency of SLIT with* Dermatophagoides farinae* drops between monosensitized patients and polysensitized patients (Table 3) were analyzed. Based on *α* = 0.05, there was no significant difference in curative effects between the two groups (*P* > 0.05), which meant that SLIT with* Dermatophagoides farinae* drops improved nasal symptoms to a similar degree in both monosensitized and polysensitized patients. In a previous study, Malling et al. [12] performed immunotherapy using a single species grass vaccine and demonstrated that it was equally effective in polysensitized and monosensitized subjects.

### 3.4. Cross-Reactivity Analysis of Dust Mite Allergen

**Figure 2:**
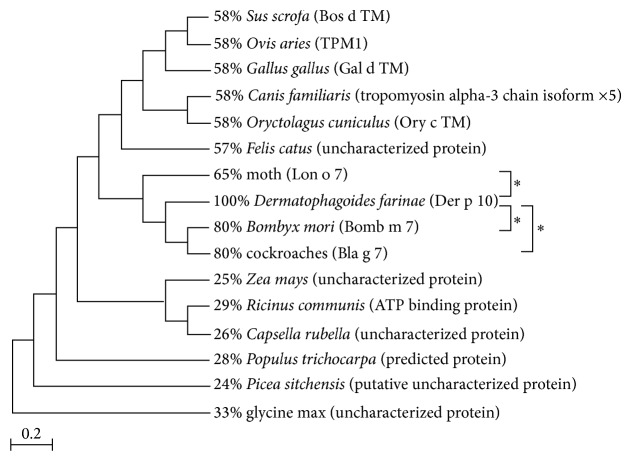
The phylogenetic tree (N-J method) and amino acid identity between Der p 10 and other allergens used for SPT.

83% of the polysensitized patients allergic to dust mite were also allergic to cockroach (Table 2). Previously, many researches have reported that dust mite allergen is highly cross-reactive with other allergens. Therefore, we considered the cross-reactivity of dust mite allergen with other allergens during our analysis. Here, we used bioinformatics methods to explore common identities among dust mite, cockroach, silk, and other allergens. The phylogenetic tree of dust mite major allergen Der p 10 and other allergens showed that identity between Der p 10, silkworm allergen Bomb m 7, and cockroach allergen Bla g 7 had reached 80% (Figure 2), and the identity between Der p 10 and moth allergen Lon o 7 was 65%. FAO/WHO experts on the allergenicity of foods [13] advise that cross-reactivity between food allergens has to be considered when there is more than 35% identity in the amino acid sequence of the allergens, using a window of 80 amino acids and a suitable gap penalty. To a certain degree, this advice might be applicable to aeroallergens like Der p 10. Because of the high amino acid identity with other aeroallergens, Der p 10 could be cross-reactive with some other inhaled allergens, which could lead to the high positive rate to cockroach, silk, and moth seen in dust mite sensitized patients.

Data from 11,355 subjects in the first European Community Respiratory Health Survey showed that 16.2% to 19.6% were monosensitized patients and 12.8% to 25.3% were polysensitized [3]. Although polysensitized patients were a large proportion of the survey, immunotherapy cannot be performed in response to every positive reaction to allergen preparations as some positive results are caused by cross-reactivity. Fortunately, our study showed that immunotherapy with house dust mite extract was equally effective in the AR subjects who were sensitized to multiple allergens when compared with the monosensitized subjects. Ciprandi et al. [14–16] have also published several reports on the use of primarily single allergen SLIT in polysensitized subjects and concluded that single allergen SLIT was safe and effective in polysensitized patients. However, a placebo effect was not considered in our study, which could potentially have caused a 1.3% increased response to SLIT according to the research [17] and, thus, double-blind placebo-controlled trials will be performed in our further studies.

In this study, we found that most dust mite sensitized patients also reacted to winter pollen, silkworm, cockroach, and moth. We further explored the relationship between dust mite allergen and other allergens using a phylogenetic tree, which showed a high identity between dust mite major allergen Der p 10 and silkworm, cockroach, and moth allergens. Therefore, we concluded that this cross-reactivity among dust mite, silkworm, cockroach, and moth allergens played a critical role in the development of AR. Dust mite extract used for immunotherapy was composed of more than 20 different house dust mite (HDM) allergens including major allergens Der p 1, Der p 2, and Der p 10. Many researchers have reported that Der p 10, one of the tropomyosin derivatives, has a high cross-reactivity with other tropomyosin allergens (Bla g 7, Pen a 1, etc.) [18–20], but in some regions such as American inner cities, France, and Italy this is rare [21, 22]. One possible explanation might be that the factors that influence cross-reactivity between mite allergens and other tropomyosin allergens are complicated and that dietary habits, living environment, and genetics all play a role in the development of multiple sensitivities and could affect therapeutic and assay results.

Resch et al. reported [23] that the patients who were positive in Der p 10-IgE tests were generally sensitive to many other allergens and, thus, Der p 10 might be a diagnostic marker for HDM allergic patients who are not sensitive to Der p 1 and Der p 2 but react to other HDM allergens. In addition, Bronnert described IgE to Der p 1, Der p 2, and Der p 10 as the markers for HDM allergy [24]. Immunotherapy with representative allergens based on cross-allergenicity is the tendency and using Der p 1, Der p 2, and Der p 10 as immunotherapy vaccines represents an attractive treatment option, especially for polysensitized patients.

## 4. Conclusion

In this study, we determined that SLIT with* Dermatophagoides farinae* drops in polysensitized house dust mite AR patients showed improvements in nasal symptoms comparable to that seen in monosensitized patients.

## Figures and Tables

**Table 1:** Standards of symptom score of allergic rhinitis.

Symptom score	Sneeze^∗^	Rhinorrhoea^#^	Rhinobyon	Rhinocnesmus
1	3~5	≤4	Conscious inspiratory	Intermittent
2	6~10	5~9	Intermittent	Formication but supportable
3	≥11	≥10	Mostly breathing through mouth	Formication and insupportable

^∗^The number of continuous sneezes. ^#^The number of times blowing nose per day.

**Table 2:** Skin prick tests results^∗^.

Allergen species	Positive cases	% positive in polysensitized patients	% positive in all patients
Dust mite	30	100.0	100.0
Animal dander	16	53.0	32.0
Spring pollen	10	33.0	20.0
Summer pollen	13	43.0	26.0
Autumn pollen	15	50.0	30.0
Winter pollen	21	70.0	42.0
Amaranth thorn	13	43.0	26.0
Cockroach	25	83.0	50.0
Moth	15	50.0	30.0
Honey bee	13	43.0	26.0
Silk	19	63.0	38.0
House dust	11	37.0	22.0
Padding	7	23.0	14.0
Cocoon filament	7	23.0	14.0
Cat hair	12	40.0	24.0
Dog hair	9	30.0	18.0

^∗^The other 20 patients are only allergic to dust mite based on the results of skin prick test.

**Table 3:** Curative effect analysis of polysensitized and monosensitized patients.

Group	Curative effect			*H*_*C*_ value	*χ* ^2^ 0.05, 1	*P* value
	Markedly effective	Effective	Ineffective			
Monosensitized	9 (45%)	7 (40%)	4 (20%)	0.1890	3.841	0.663
Polysensitized	13 (43%)	8 (27%)	9 (30%)			

Total	22	15	13			
